# The role of trust in the implementation and uptake of COVID-19 response measures: a qualitative study of health professionals’ experiences in Tanzania

**DOI:** 10.1186/s12913-023-10043-3

**Published:** 2023-10-10

**Authors:** Emmy Metta, Elizabeth H. Shayo, Frida Ngalesoni, Albino Kalolo, Kasusu Nyamuryekung’e, Innocent B. Mboya, Harrieth P. Ndumwa, Belinda J. Njiro, Maryam A. Amour

**Affiliations:** 1 Department of Behavioral Sciences, School of Public Health and Social Sciences, Muhimbili University of Health and Allied Sciences, P.O. Box 65001, Dar es Salaam, Tanzania; 2 National Institute for Medical Research, P.O. Box 9653, Dar es Salaam, Tanzania; 3 AMREF Health Africa in Tanzania, P.O. Box 2773, Dar es Salaam, Tanzania; 4 Department of Public Health, St. Francis University College of Health, and Allied Sciences, P.O. Box 175, Morogoro, Tanzania; 5 Department of Community Dentistry, School of Dentistry, Muhimbili University of Health and Allied Sciences, P.O. Box 65001, Dar es Salaam, Tanzania; 6 Department of Epidemiology and Biostatistics, Institute of Public Health, Kilimanjaro Christian Medical University College, P.O. Box 2240, Moshi, Tanzania; 7 Department of Translational Medicine, Lund University, 202 13 Malmö, P.O. Box 50332, Malmö, Sweden; 8 Department of Community Health, School of Public Health and Social Sciences, Muhimbili University of Health and Allied Sciences, P.O. Box 65001, Dar es Salaam, Tanzania

**Keywords:** Trust, COVID-19 responses, Interpersonal trust, Health-system trust, Stigma, COVID-19 vaccines

## Abstract

**Background:**

Even though trust is placed at the central point in ensuring proper functioning of the health systems, studies remain scant on how it affects both the implementation and uptake of COVID-19 response measures in low- and middle-income countries such as Tanzania. This study, therefore, explored the role of trust in the implementation and uptake of recommended COVID-19 response measures including vaccines from the perspective of health professionals in Tanzania.

**Methods:**

This cross-sectional qualitative study was implemented in four of Tanzania’s thirty-one regions. Qualitative data was collected through 26 in-depth interviews held with regional and district disease outbreak response teams, district cold chain co-ordinators and health facility in-charges. In addition, five focus group discussions and seven group interviews were conducted with healthcare workers from the lower-level health facilities. Thematic analysis was conducted and applied the trust constructs.

**Results:**

Interpersonal trust and health system trust emerged as two major themes in the study. Interpersonal trust was reported to stem from lack of transparency that instigated fear, worries, and confusion regarding the implementation and uptake of the recommended response measures. The distrust was mainly between health professionals in health facilities and those assigned to isolation centres as well as between patients and community members. On the other hand, the health system trust was shaped by mixed feelings regarding COVID-19 vaccine national decisions, and conflicting messages from national officials, politicians and religious leaders on COVID-19 responses, safety, and effectiveness of the vaccines. Questions surrounding the short duration of clinical trials, indeterminate post-vaccination protection duration, impotence-linked beliefs, freemasonry notion and unclear vaccinated cards information are other reported contributory factors to mistrust in the health system. However, after a comprehensive health education and experience in COVID-19 vaccination administration most professionals affirmed the effectiveness of the vaccines in limiting infections and its severe consequences.

**Conclusion:**

Participants indicated limited trust at both interpersonal and health system levels aggravated by lack of transparency, unclear and conflicting messages on COVID-19 infections and response measures. Enforced transparency on pandemics alongside standardised messages from the reliable sources is crucial in enhancing trust in implementation and uptake of the recommended response measures.

**Supplementary Information:**

The online version contains supplementary material available at 10.1186/s12913-023-10043-3.

## Introduction

The coronavirus disease 2019 (COVID-19), which was first reported in Wuhan, China at the end of 2019 spread across the world at an alarming speed. The World Health Organisation (WHO) officially declared it a pandemic in March 2020. In its first wave, most of the countries in sub-Saharan Africa reported a spiralling number of cases, both imported and locally- acquired. To-date, the pandemic has affected many people in all spheres of life in addition to overburdening the health systems in afflicted countries [1, 2]. COVID-19 was first reported in the United Republic of Tanzania (URT) on March 15th, 2020, in Arusha region. Subsequently, the infection spread to different parts of the country. In response, the Tanzania government adopted various WHO-recommended self-protective measures and partial lockdowns including school closures to limit and contain transmissions [3]. The government also established specific centres for treating confirmed COVID-19 cases and for isolating individuals suspected to be infected for 14 days. By July 2021, the Tanzania government launched a nationwide COVID-19 vaccination campaign prioritising frontline healthcare workers, older people, and individuals with co-morbid conditions. However, as more vaccines arrived into the country all age groups from 18 and above benefited from these jabs.

The COVID-19 preventive measures including vaccines are evidence-based recommendations for controlling the COVID-19 pandemic. The uptake of these recommendations largely depends on public trust in the messenger (in this case public officials and other influential public health actors) and the message delivered by the messenger. The degree and quality of public trust in the government, public health agencies, pharmaceutical companies (manufacturers), the wider health systems and the science behind what is advocated can influence either negatively or positively the willingness of individuals and communities to adhere to the control measures and uptake of the COVID-19 vaccines. Implicitly, trust level depends on how individuals build confidence in science and the competence of a person, organisation or institution promoting science related knowledge or its products.

Trust in COVID-19 preventive measures and vaccines is epistemic in the following ways:1) Trust in the measures instituted or the vaccine based on its safety and quality; 2) Institutional trust occurs in cases where preventive measures and products or vaccines come from (institutional affiliations, organisations and their reputations); and 3) Inter-personal trust stemming from the person who recommends preventive measures or vaccine (recommendations by healthcare workers, neighbours, relatives and peers) as well as the nature of recommendations whether positive or negative [4]. Additionally, an interaction between a person and an institution can lead to institutional trust built over the years based on the knowledge, competence, and skills that the state, institutions, or healthcare workers bear. Default asymmetry in information, comprehensibility, and power between the vaccine providers and vaccine recipients make persons who decide on a vaccination vulnerable since they also invest to some faith in the trusted party.

Overall, trust is one of the ultimate tests of either success or failure of a given health system. It also depends on the health system’s responsiveness to the population’s needs and demands, which in turn affects the satisfaction level among patients. Adequate delivery of quality services and acceptable access to prioritised services are key determinants of responsiveness [5]. Furthermore, trust is a relational notion that generally occurs among people, between people and organisations, and between people and events. Trust can also be both faceless and face-work [6]. Faceless refers to the fabric of society and systems (including health systems) whereas face-work describes the interpersonal dimensions that exist among people. As such, trust relations cultivated through inter-personal interactions are crucial in sustaining system-level trust, transforming and enriching patient experiences and related health outcomes [7]. Similar to many other pandemics, the curbing of the COVID-19 would significantly be influenced by the widespread uptake of the recommended response measures. Indeed, for effective enhancement of the COVID-19 response measures, studying issues of trust from a broad-based perspective and its influence pertaining to the compliance to different policy and practice recommendations is crucial.

Notably, a few months after the government had launched COVID-19 vaccination campaign, low uptake was evident among the priority groups including health professionals, hence the need to explore the extent to which trust played in determining the enhancement of the utilisation of COVID-19 vaccination services and other preventive measures. The study therefore explored trust the government displays in being receptive to international recommendations, trust in institutions and people introducing such recommendations, the trust the public have in government officials and other public health actors calling for implementing these anti-COVID-19 measures. To the best of our knowledge this is the first study to describe how trust shaped the implementation and uptake of COVID-19 responses in Tanzania.

## Methods

### Study design and settings

This paper reports the findings of a cross-sectional mixed methods study that was conducted in seven of Tanzania’s thirty-one regions, targeting healthcare workers operating in public health facilities. The qualitative data was collected in four purposively selected regions of Dar es salaam, Simiyu, Kilimanjaro, and Tabora, which had outstanding performance in fostering the COVID-19 vaccination uptake amidst COVID-19 prevalence. From each of these regions, we purposively selected one district for the study based on the districts’ vaccination uptake rate. Besides a regional hospital, we also picked a district hospital and two health centres from each district for the study to be comprehensive and to be representative of the different levels of healthcare and cadre.

### Study population and sampling

The study population comprised representatives of regional and district outbreak response teams, regional and district cold chain co-ordinators, health facility in-charges, and healthcare workers. The health workers comprised nurses, clinicians, laboratory personnel, pharmacists, and attendants. The recruitment of participants by virtue of their roles in COVID-19 preventive measures and vaccinations also ensured diversity in cadres, settings and levels to gather rich information on issues of trust related to implementing measures and vaccinations in different situations. At the regional level, regional medical officers (RMOs), regional immunisation and vaccination officers, and medical officers in-charge of regional hospitals and selected healthcare workers participated in the study. Similarly, at the district level, district medical officers (DMOs), district immunisation and vaccination officers, medical officers in-charge of district hospitals and selected healthcare workers partook in the research. At the health centre levels, the participants were health centre in-charges and selected healthcare workers. All these participants from different levels and cadres participated in either in-depth interviews, focus group discussions, or group interviews. Table 1 presents the distribution and number of data collection activities in each study region.

**Table 1:** Number and type of data collection activities per study region

Data collection Technique	Dar es Salaam	Kilimanjaro	Simiyu	Tabora	Total
In-depth interviews	8	6	5	7	26
Focus Group Discussions	1	1	2	1	5
Group Interview	2	2	0	3	7

### Data collection

Data collection at different levels of the healthcare system involved in-depth interviews (IDI), using a developed IDI guide, held with key informants at regional and district level from epidemic response and vaccine committees made up of regional and district medical officers and vaccine co-ordinators. The key informants also included in-charges from health facilities administering COVID-19 vaccines at regional and district hospitals. These IDIs generated detailed information on the key informants’ experiences with executing COVID-19 recommended measures and understanding how issues of trust informed their practices. Whereas the focus group discussions (FGDs) were conducted at hospitals, group interviews were held at health centres. Each FGD at hospital had 8–12 members. The group interviews at each health centre comprised 2–4 care providers. Frey et al. stated that group interviews differ from traditional face-to-face interviews because they involve more than two persons interviewed spontaneously in the same location [8]. Both FGDs and group interviews enabled the study gain a broader understanding of the participants’ trust and how it shaped their practices in the implementation and uptake of COVID-9 recommended response measures. Topic guides facilitated discussions both the FGDs and group interviews.

Trained research assistants in collaboration with the authors collected the data. All data collection guides were pre-tested with the results informing further improvement of the final versions for administration in the study. After obtaining their informed consent, we assured the participants of anonymity and confidentiality. All the interviews and FGDs were conducted in Kiswahili and audio-recorded with prior permission of participants. On average, the duration for in-depth interviews were 45–55 min, group interviews 30–45, and focus group discussions 60–75 min. We applied the principle of bracketing to ensure our pre-understanding information did not affect the data. Moreover, we set aside our repertoires of knowledge, beliefs, values and experiences to describe accurately the participants’ life experiences [9]. Furthermore, we maintained and reviewed field notes as a reflective diary during the analysis to enhance reliability. The interviews and the discussions were held in places that ensured maximum privacy and clear recording.

### Data analysis

The audio-recorded interviews and focus group discussions were transcribed verbatim. These transcripts were reviewed by both the first and second authors against the audio recordings for quality control purposes. Re-reading of several transcripts produced different codes that all authors discussed for consensus before the actual coding. We followed the five stages of thematic analysis, as described by Braun and Clarke [5], to establish meaningful patterns in the data: Familiarisation with the data, generating initial codes, searching for themes among codes, reviewing themes and, finally, presenting the results. The coding also helped to identify illustrative verbatim quotations covering various themes in accordance with the study objective.

## Results

### Characteristics of the participants

A total of 100 individuals participated in the study made up of 26 Key Informants and 74 participants drawn from health care facilities. Whereas the former group took part in IDIs the latter participated in five FGDs and seven group interviews. There were 54 male and 46 female participants, mostly holding a diploma in clinical medicine qualification. Their cadre varied, majority comprised of nurses, followed by medical doctors, and laboratory technicians.

### Types of trust relative to COVID-19 interventions

The study results indicate varying types and patterns of trust, among and between study participants that can be categorised into two main dimensions. The first dimension, “interpersonal trust”, was characterized by the trust in national officials and the WHO recommended COVID-19 measures, the trust among health professionals and patients and the trust between health professionals and the community. The second dimension, “healthcare systems trust” was characterized by the trust in decisions to use COVID-19 vaccines and their effectiveness and the trust in sources of information.

### Interpersonal trust

#### Trust in national officials and WHO-recommended COVID-19 measures

The key informants at both regional and district levels indicated lack of trust in national officials and raised concerns, worries and confusion about the official measures for curbing COVID-19. This lack of trust stemmed from limited clarity on what constituted trustable measures due to competing interests among politicians, scientists, and religious leaders that often clouded what the people needed to embrace as the truth. In this regard, the informants cited the initial country’s responses to COVID-19 that lacked transparency not only about the tally of confirmed cases and resulting deaths but also about how the disease was transmitted. They also noted that the officials made COVID-19 related information so confidential that even where the data existed the awareness was restricted only to the authorities. In addition, healthcare officials reported being prohibited from sharing COVID-19 information with their patients or community members. Such restrictions persisted even during the third wave of the pandemic, as one of the key informants explained:

*“…even now I have the data for COVID-19 cases in our region but since I have no authority to share, I cannot share it” (IDI02)*.

Limited trust in national officials also emerged during group discussions with healthcare workers. These participants reported that the government’s lack of seriousness and transparency in its response to the COVID-19 pandemic challenged their trust. In fact, healthcare workers were also not allowed to indicate COVID-19 in their diagnosis; instead, they recorded any other respiratory condition such as pneumonia. Based on their testimonies, this questionable practice inevitably demotivated the health professionals and, doubly so, since they were also forced to misinform—in short lie to—patients and their relatives about the prognosis:

*… sometimes even if a patient is diagnosed with COVID-19 we were not allowed to say it rather we used to say it is pneumonia and the patients’ relatives kept on attending the patient as a normal case* (GI05).

The healthcare professionals further reported about frustrations, especially when their professional knowledge on the prevalence of the pandemic in the country was at odds with misleading statements of government officials. This situation impacted on their level of trust and contributed to the knowledge practice gap between what the health professionals knew and trusted and what the government directed them to practice.

*We knew for sure there were COVID-19 patients in the country, particularly from our region but we were not allowed to tell the truth about the disease, so we ended up disappointed since COVID-19 kept on affecting us and the number of healthcare providers who contracted it was rising daily* (GI02).

In many cases, the political leaders reportedly elevated the spread of misinformation and sowed seeds of confusion pertaining to the pandemic and its responses. Yet, communities placed their trust in these same leaders. The health professionals recounted that it was difficult for them to admit patients and inform them or their relatives about the COVID-19 diagnosis because of widespread and politicised denials that there were no COVID-19 infections in the country. The restrictions to misinform people about the COVID-19 situation made it hard for care providers to handle such patients.

Commonly, the study participants reported that during the first and second waves of COVID-19, citizens’ trust in herbal medications and divine intervention through prayers as the effective means for ameliorating COVID-19 symptoms. This trust, however, evaporated during the third wave of the epidemic which coincided with a change in national leadership, which implored people to get vaccinated and toned down on the epidemic denial rhetoric. The rapid change in approaches and responses to COVID-19 brought a dilemma not only for the healthcare professionals but also for community members generally already conditioned to trusting in containing COVID-19 by taking herbal concoctions and fervently reciting prayers.

#### Trust among health professionals and patients

Healthcare providers including key informants at the regional and district levels reported cases of distrust among their ranks, especially when it came to those directly caring for COVID-19 patients. This lack of trust eroded work efficiency since the healthcare providers found with any symptom suggestive of COVID-19 such as flu had to remain quarantined at home until the signs dissipated. Besides, providers who worked in isolation wards reported dreading they could have contracted the disease even in the absence of such proof. Lack of trust in their safety and dread for being infected reportedly further cultivated mistrust and misunderstandings among care providers as the following testimony illustrates:

*It caused several misunderstandings among healthcare providers. Those who worked in isolation wards were so stigmatized that every provider tried to avoid them since everyone worried about one’s safety/ life and family because we already heard about the number of healthcare providers who were dying in the world due to this disease* (GI01).

Lack of trust was also reported between healthcare providers and patients who visited health facilities. The health professionals explained that, on the one hand, the patients had lost trust in visiting health facilities for fear of being infected; and, on the other hand, the healthcare providers did not trust patients and became hesitant to attend to them. In other words, both parties cast suspicious looks at the other party as a possible COVID-19 carrier. Healthcare providers feared to be at a greater risk of contracting the disease from their patients than others due to the nature of their work. To attend their patients well and take detailed patient history, they had to be near the patient, something that tested their compliance with the recommended COVID-19 response measures. It also emerged that in many cases, the health professionals attended to COVID-19 patients before diagnosis. Explaining the context that affected the trust between patients and healthcare providers, one health officials said:

*… we meet patients directly and we attend to them even before we diagnose them to determine whether it is a COVID-19 case or not… that way I must be at risk because attending patients requires you to stay near them, listen, touch [mm] so it is very difficult to keep social distance when attending to a patient, and you find some of these patients are seriously sick and they cannot wear masks… some cough so it is all about risks for us because even if say I wear masks still patients can transmit the viruses on the table through coughing and later on I may touch the table, so it is a very risky environment* (IDI07).

Lack of trust due to safety concerns also jeopardised the quality of the healthcare services delivered to the patients. In fact, when the healthcare providers thought of their colleagues who had died due to COVID-19, their confidence went down dramatically. As one of them explained:

*The quality of care was poor since we had to think about our safety. Sometimes you keep such a social distance that you don’t follow all the procedures you were supposed to carry out with the patient because you attend to the patient while seriously thinking about those providers who were dying. Then, you become so worried. Generally, the quality of care we provide has been poor during this COVID-19 period. We sometimes ignore the SOPs just to make sure that we remain [hypothetically] safe* (FGD01).

Lack of trust reportedly instigate stigma and humiliation of the patients:

*There was a time at the pharmacy when I had wished I could throw the medicines to patients instead of giving them in their hands because I was so worried after I heard about the number of doctors and nurses who had died, especially during the third wave* (Group interview, GI06).

Such testimonies suggest how deeply the fear of contracting the disease was so engrained among healthcare providers that they were tempted to neglect duties associated with taking care of COVID-19.

#### Trust between health professionals and community members

As the COVID-19 prevalence escalated, care providers declared a declined trend of trust from their fellow community members. This discrimination was particularly much more evident with those who were assigned to work at isolation centres whenever they returned home where they found their household members treating them as positive cases, hence their being stigmatised for COVID-19:

*Sometimes, when we arrive at home from work, the moment we reach at the gate family members start to shout at us, ‘Hey change your clothes! Take a shower!’ because they know where we come from but we ask them not to stigmatise us since we know how to take care of ourselves and of them so it is a challenge* (GI04).

Fear of contracting the disease from a family member, who is a care provider also lowered trust based on evidence from interviews and discussions. One of the participants said:

*… there was a time I started thinking maybe it will reach a point when they [family members] will ask me to remain at the gate and not allow me to enter the house. Sometimes, I may buy a gift for my family on my way back home but when I arrive at home, they refuse even to touch it. They used to tell me ‘Don’t touch anything, don’t touch the door lock, put your clothes in the bathroom’ so it was some sort of stigma* (GI03).

The healthcare providers, who worked in the isolation units, also reported being called and labelled different names by community members such as *“Watu wa Corona”* (literally meaning COVID-19 people) simply because they worked in health facilities where they attended to COVID-19 patients. Similarly, another participant said:

*There is a huge difference when it comes to how people treat not only me but also other healthcare providers during this period of the COVID-19 outbreak. One day, I was in one of the facility’s ambulances, and we stopped somewhere. When we alighted from the car, people started to shout at us ‘Corona! Corona!’ Since the outbreak of this disease people associate healthcare providers with COVID-19. Thus, they know we are in the front line. Sometimes, they call us “Watu wa Corona” [COVID-19 people]* (IDI03).

With the introduction of COVID-19 vaccines into the country during the third wave as a result of change of political regime, community members indicated hesitancy and limited trust in healthcare providers and, at times, treated them as traitors. However, with the intensive education and awareness creation activities on the usefulness of the vaccines many community members started trusting health professionals and realised they could help prevent the disease transmission and ease its severe effects. The heightened trust in health professionals also resulted in increased consultations with the people since they emerged as reliable role models and experts:

*Most of the community members and health professional have been treating me as a person who is well-informed about health-related issues. They have been coming to me to seek information on the COVID-19 pandemic since they know that I am the right person who can give them the right information* (IDI01).

**Fig. 1:**
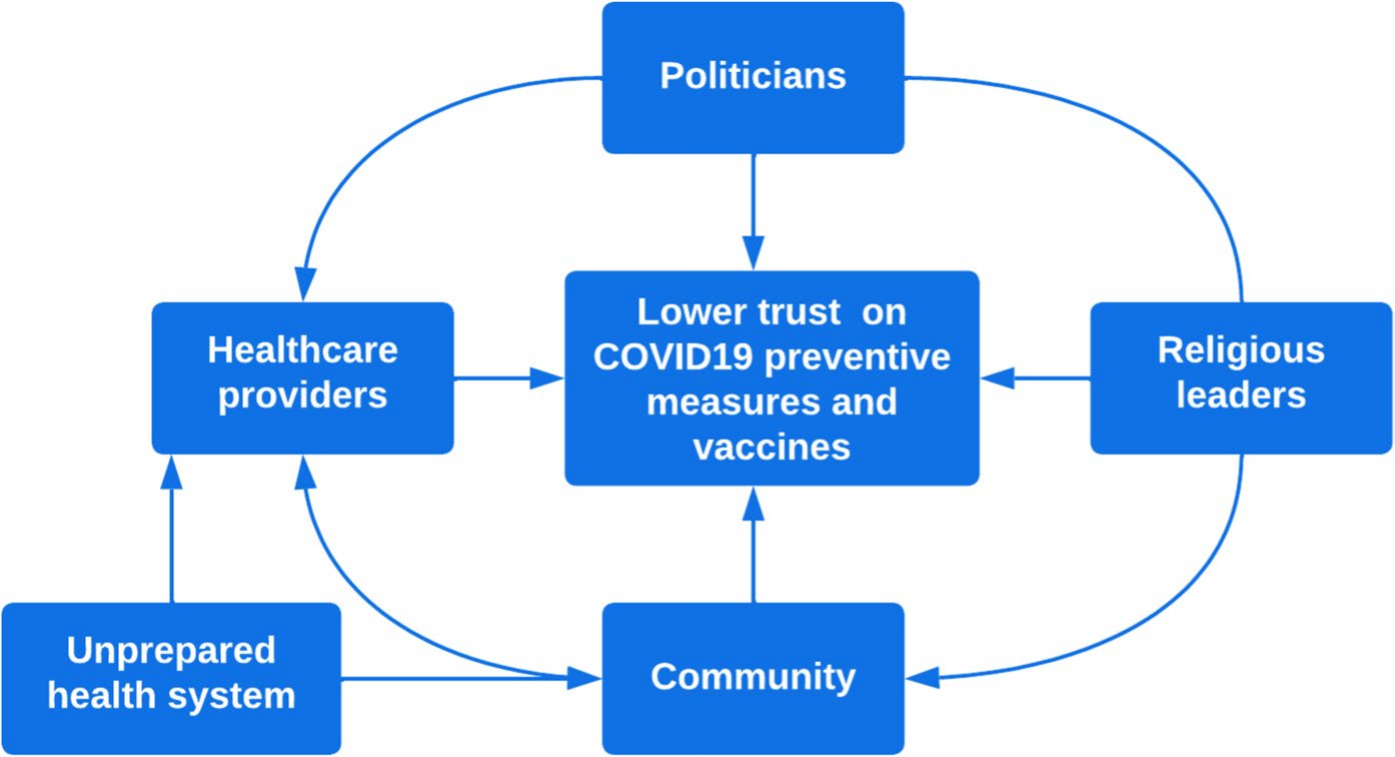
Cycle of mistrustful at interpersonal relations and health system dimensions

Trust seems to operate in a vicious cycle, with each element influencing the other, as Fig. 1 illustrates:

### Healthcare system trust

#### Trust in decision to use COVID-19 vaccines and their effectiveness

Some health professionals, mainly leaders including regional and district medical officers and co-ordinators of vaccine distribution, reported trust in the government decisions to use COVID-19 vaccines in the country. Since they were prioritised in receiving vaccination, they were happy and proud of being respected by the government for the privilege. These health professionals reported that they had confidence in vaccines since none of the vaccinated individuals in other countries had experienced any adverse effects. The following account illuminates on the healthcare workers’ opinions on COVID-19 vaccinations and their being prioritised for such jabs in the country:

*A health worker is like a soldier in battle who should protect himself and others… I personally thought it was a right decision to deploy vaccines in the country and I feel like the government prioritised us because wanted to protect its health professionals against this disease since they are working under risk environment* (IDI010).

On the other hand, healthcare workers, especially those working at lower-level health facilities, reported mixed sentiments regarding the vaccines. Whereas some were concerned about the vaccines, others said that the use of vaccines in the health facilities led to reduced number of new COVID-19 infections, admissions and severe cases. In addition, there were few deaths observed among vaccinated people and, in some regions, none. One of the health professionals at the regional level further explained during an interview:

*We track and make close follow-up of those who are vaccinated, some of whom do not even wear masks or maintain social distance but have not been infected and we don’t have any reported death of vaccinated and this can be used as evidence that the vaccines that are now available in the country are effective* (IDI015).

The health professionals said that vaccines contributed to reduced costs of healthcare service provision in the country including for buying oxygen cylinders, protective gear, and drugs needed in bulk for critical ill-patients. Other positive accounts of trust in the effectiveness of the vaccines reported in the study are as presented in Table 2:

**Table 2:** Professional accounts of trust on effectiveness of COVID-19 vaccines

▪ Vaccines reduced the number of deaths and hospital admissions.	
▪ Vaccines reduced the burden of the disease to the hospitals and the need of purchasing bulk of protective gears, treatments, and equipment to accommodate a huge number of COVID-19 patients.	
▪ Vaccines cleared fears and created confidence and peace of mind among health providers and community members as now they can interact and contact with less or no doubt.	
▪ No complaints or patients brought to the hospitals in serious condition due COVID-19.	
▪COVID-19 vaccines provide additional antibodies against the virus and strengthen the body immunity in fighting the disease.	

Personal experience with the vaccines informed the professionals trust in their effectiveness:

*I used to suffer from flu, which ended after I received COVID-19; this is not only me, but many people provide testimonies on this aspect. As we speak, there is none of our patients in the hospital who had received the COVID-19 vaccine admitted suffering from the disease” *(IDI09).

Conversely, the healthcare professionals, who raised concern about the COVID-19 vaccines had several reservations attributable to the lower level of trust such as lack of information on vaccine quality assurance, effectiveness, side-effects, and time that was spent on developing them. The mistrust was also informed by the absence the expiry date, duration of post-vaccination protection, and existence multiple vaccines.

Discussions with the key informants revealed that the information communicated by some leaders through the social media on the vaccines also created mistrust in the COVID-19 recommended responses:

*Health workers including myself felt uncomfortable because of the explanations provided by one of the religious leaders [name withheld]. Of course, most people were rigid about taking COVID-19 vaccine and up to now some healthcare personnel are not yet vaccinated; the message from [name withheld] was strong to the extent that any person with brain could believe and agree with him* (IDI012).

Negative accounts of trust in the vaccines were linked to the country of origin which raised questions about the reliability of the manufacturer. In this regard, participants reported lack of confidence in vaccines from China such as Sinopharm as they doubted their efficacy. Further questions arose based on the rather short duration of COVID-19 vaccine trials before their release for universal use. Participants also raised concerns about possible COVID-19 vaccine side-effects due to rumours that they might interfere with their DNA system. Some rumours presented these vaccines to have an embedded ‘chip’ that once in the body of a vaccinated person serves monitoring purposes. The following statement came from one of the health providers during an FGD:

*Previously, we used to do steaming, but today you want us to take COVID-19 vaccines, why? This I don’t understand, we don’t know and not sure about COVID-19 vaccines, because no training has ever been provided. It has neither an expiry date nor side-effects. the JJ card shows two spaces for vaccines 1 & 2 but we provide only a single shot…so people do ask,…‘ You told us that we will be injected only once now why two spaces? Shall I repeat to complete a dose! How long will COVID-19 vaccines last after getting the injection? Some people think that COVID-19 jabs are just trials; others want to know whether if they that kind of vaccines they would be allowed to enter country X. Even most of us have not yet taken COVID-19 vaccines because we don’t understand it!* (FGD03).

Similarly, during group interviews, one of the participants said:

*The COVID-19 vaccines are on trials; it is a business thing. Why do implementers persuade us very strongly to be vaccinated? What is the hidden agenda? Why should people get registered electronically using the national identity number, what is the importance of being vaccinated while we are told that one may still get infection? …Youths do not feel part of the COVID-19 vaccines exercise because they see themselves strong and have no chronic diseases such as Blood pressure, and diabetes* (GI07).

These professionals further reported that this mistrust about COVID-19 tricked down to the community level and, particularly, because leaders had been misinformed about the vaccine, hence their failure to convince others to get vaccinated.

#### Trust in the source of information

Development of trust was dependent on the source of information. A trustable source of information included medical professionals, government leaders, especially the President, and the Ministry of Health, mainly the Chief Secretary. In this regard, the US-based, Centre for Disease Control (CDC) emerged to be an additional credible source for reliable information. The trust in information sources was linked to reasons as indicated in the Table 3:

**Table 3:**
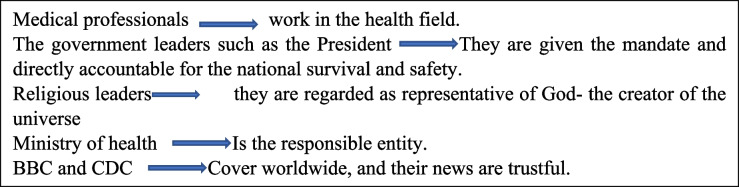
Professionals accounts on the reasons of trust on information sources

## Discussion

The study findings have revealed variations in levels of interpersonal and health systems trust on COVID-19 response measures including vaccines. The initial government and political officials’ communications about COVID-19 and its control measures instigated worries and confusion among health professionals and other stakeholders, which ultimately lowered the level of trust. Mistrust was further amplified by conflicting COVID-19 messages communicated to the health professionals and to the public by government officials, scientists and religious leaders. On the other hand, the misinformation about COVID-19 trend delivered to the public affected the health professionals’ practices, some of whom failed to abide by the ethical code of conduct in diagnosing and treating COVID-19 patients or suspected cases. The resulting chain of mistrust between Government-politicians-health professionals-community members compounded the COVID-19 problems and difficult to manage. Yet, proper functioning of health systems requires trust in healthcare as one of the central processes, which is vital in the provision and utilization of health care services [10]. Trust also plays a crucial role in fostering people’s interaction with the system and the outcome of that interaction [11]. In the context of the ongoing COVID-19 pandemic, trust is an important determinant in the implementation and uptake of recommended measures.

The restrictions exerted on providers in the study not to disclose COVID-19 diagnosis to patients due to political interference, pressure to work with vaccines with partial/no evidence of their efficacy, working in unsafe conditions and without patients’ trust and vice-versa challenged the providers’ professional ethics and conduct and could even undermine their best practices essential in improving their patients’ safety and care outcome [12]. In fact, both the rights of patients and health providers could suffer as a result. One solution is to keep politics out of professionalism by empowering and protecting health professionals to practice according to their ethical codes of conduct.

Indeed, the erosion of trust between health service providers and politicians influenced the decisions of the former group in responding to COVID-19 preventive measures. Inconsistency and lack of consensus between different information sources also reportedly influenced how providers responded to the COVID-19 pandemic in different settings [13]. Recent evidence further hints at how widespread misleading information was with more than a quota of the most viewed YouTube videos on COVID-19 containing misinformation or inaccuracies [14]. Overall, the consequence of misinformation goes beyond the physical and psychological impacts because confusions can trigger fear-based actions [15] particularly when the information came from trusted sources or authorities.

Since a successful implementation of public health interventions depends on the public trusting the healthcare professionals and the healthcare system in general [16, 17], the widespread distrust the study has established during the pandemic remains a source of grave concern. Worries about contracting corona viruses amplified the situation both among the providers and the patients. Such fear of catching COVID-19 and feeling of helpless among healthcare providers has also been reported elsewhere [3, 18, 19]. The fear was also exacerbated by limited availability of personal protective gears against COVID-19, forcing some care providers to dread attending to the sick. Lack of trust also contributed to reduced work efficiency among healthcare workers.

Whereas patients feared visiting health facilities to avoid ending up in isolation centres, worries about such seclusion similarly persisted among healthcare providers suspected to have COVID-19 related symptoms as recommendations for the management of chronic respiratory diseases during the COVID-19 epidemic reveal [3]. The isolation of healthcare workers did not only strain the healthcare system already overstretched by a shortage of human resources for health [20] but also challenged the social, mental and psychological health of the healthcare providers [21]. Historically, social isolation means “an objective state of having minimal social contact with other participants” [22]. Although this study did not dwell on these issues in detail the need to identify the perceived consequences of social isolation on healthcare providers and health services provision cannot be overstated.

The study also found that mistrust between healthcare professionals and community members that informed the social stigma and discrimination practices against those believed to have had direct contact with COVID-19 patients. Frontline providers reported experiencing such stigma and discrimination not only from their fellow providers but also from their family and community members. Similar observations were also reported across the globe [19, 23, 24]. Mistrust of healthcare workers shaped the practices of families and community members of insulting and imposing restrictions as was also the case in Egypt [25], Malawi, India, and Mexico [23].

Indeed, stigmatisation against pandemics is not a new phenomenon [24] and for emerging pandemic conditions such as COVID-19 characterised by several mythologies, stigmatisation of the frontline health providers and patients is not surprising [24]. Stigma, according to Mohammed et al., refers to “any social or physical trait or gesture that disqualifies an individual’s social identity, such as disqualifying them from full social acceptance” [26]. Stigmatising healthcare workers may have negative effects not only on their mental and psychological health but also on their morale and quality of care they provided as it has been documented elsewhere [27].

Evidence also shows that trust in government decisions and in healthcare is important in fostering the uptake of health recommendations [28]. In addition, trust and confidence in government establishments including policy-makers is crucial in vaccine decision-making [28–30]. In this study, health professionals had mixed views about their trust in the recommended COVID-19 vaccines. Many of them indicated trusting the government’s decisions and were willing to get vaccinated since they believed in the jab’s effectiveness. This trust was further strengthened not only by their own experience after being vaccinated but also the fewer numbers of COVID-19 cases and fewer admissions and deaths linked to COVID-19 that they observed after the vaccinations started in earnest. This positive development could further inform the ongoing efforts of encouraging the uptake of COVID-19 vaccines among healthcare workers and the public by citing the changes observed in terms of patient load and severity as motivations. Trust in government authorities was also associated with high uptake of the recommended COVID-19 protective measures including being vaccinated in Australia [31] and United States [30]. Information on better ways to strengthen trust in government decisions is crucial for enhancing acceptability and uptake of the health recommendations, especially during epidemics requiring prompt decisions and actions for prevention and control.

Whereas trust in government authorities induces the uptake of the recommendations, mistrust is associated with hesitancy and delayed adaptation of the recommended measures [29, 32]). Some of the health workers reported to resist COVID-19 vaccines due to rapid changes in government recommendations against COVID-19 responses because of concerns over inadequate information on vaccine quality assurance, effectiveness, side-effects, and the time spent on their development. Other studies also reported concerns about side-effects, short vaccine development, doubts about the efficacy of the vaccine and the general lack of trust, hence their unwillingness to get vaccinated [33–36]. Yet, health professionals are key personnel in delivering health services including health messages and their contacts with people could encourage appropriate health-seeking behaviour including vaccines [37]. The hesitance observed regarding vaccines in the study raises concern because of the role professionals play in vaccination campaigns and other clinical interventions.

### Strengths and limitations

Overall, to our knowledge, this is the first study that has provided useful information aimed at broadening the understanding of the role trust in the implementation and use of COVID-19 control measures among healthcare professionals and other stakeholders. The inclusiveness of healthcare providers and from different health system levels and from different regions enabled the study to garner a comprehensive understanding of the aspects shaping providers trust regarding the COVID-19 recommended responses and the context that informed their practices. However, as the study was restricted to particular group of people, healthcare professionals, who were purposively selected, hence, the results can only be generalised to conditions obtaining in a similar context. The study results can also provide useful insights aimed to inform efforts for improving trust, implementation and uptake of health recommendations during pandemics.

## Conclusion

Overall, the study has revealed critical information on interpersonal and health systems trust during the implementation of the recommended COVID-19 response measures. Trust among and between healthcare providers, patients and health officials was shaped by worries attributable to conflicting messages and misinformation on the uptake of various recommended preventive measures. Limited availability of protective gears and comprehensive education on the preventive measures impacted on both the healthcare providers’ fear to attend to patients and the patients’ fear to access healthcare services from the facilities. Politicians and religious leaders’ messages amplified the mistrust in COVID-19 vaccines. These findings call for joint efforts in the fight against the COVID-19 pandemic to provide standardised messages on COVID-19 preventive measure. This has the potential to boost trust and enhance the implementation and uptake of the recommended COVID-19 response measures. Significantly, religious and political leaders should first be sensitised on proactive messages to gain a common understanding before delivering them to the community members. The findings also call for health systems preparedness for future pandemics.

### Supplementary Information

**Additional file 1.**

## Data Availability

The datasets generated and/or analyzed during the current study are available from the corresponding author on reasonable request.
