# Supplementary material for: The role of trust in the implementation and uptake of COVID-19 response measures: a qualitative study of health professionals’ experiences in Tanzania
Source: BMC Health Serv Res. 2023 Oct 10;23:1077. doi: 10.1186/s12913-023-10043-3 (PMC10566036; doi:10.1186/s12913-023-10043-3)
Supplement: Supplementary file 1 — Additional file 1. [file 12913_2023_10043_MOESM1_ESM.pdf]

Appendix 1

IN-DEPTH TERVIEW GUIDE FOR HEALTH FACILITY INCHARGES, DISTRICT AND REGIONAL HEALTH MANAGERS

Instructions (Interviewer)

a) This tool should be used as a guide to facilitate discussions with the health facility in-charges, district and regional level health managers and should be used only after obtaining consent from the potential participant
b) Please fill in the bio-data matrix before commencing the discussion such as age, marital status, occupation/ profession, position, educational level in the different sheet provided.

1. What are your main roles in this facility/district/region? (Kindly explain if there are additional roles as a result of covid19 and its vaccine in the country)
2. Based on your experience, how can you describe the covid19 waves from 2020? Probe: What is your reflection based on the trend that you are observing?
3. How vulnerable do you see yourself for getting infected with covid19? Probe: if ever got infected with corona virus
4. Tell me what you think on the likelihood that you could transmit COVID-19 to your patients?
5. Being a health care worker/health manager, how are you usually treated by others in the community? Probe: Have you noticed anything different in how you're treated since the pandemic?
6. As you know, there are different COVID-19 vaccines in the world and in Tanzania in particular. What have you heard about the COVID-19 vaccines?
   Probe:
   a. Heard of anything that worries you?
   b. What type of worries and how they reach you?
   c. What was the source of information? (how credible the sources are?)

What can you comment as positive side about the vaccines that are currently available in the world (probe for different types of vaccines; How do you think about the vaccines that are available now?) Relate back to perceived COVID-19 risk, and how important it will be in protecting other

7. What are your thoughts about the safety of the COVID-19 vaccines? (probe different types)
8. What are your thoughts about the effectiveness (probe different types)
9. What were the responses of care providers when they heard of the availability of vaccine? (probe how did health workers reacted after hearing that they were listed as a priority group to be vaccinated?)
10. As the manager/in-charge in this region/district/health facility, what are the general response of the peoples towards the vaccines?
    i. Non health professionals
    ii. Community members
    iii. Probe: what do you think influences their responses

Generally, what are the misconception on vaccines by stakeholders at different levels

- Community members

- Health professionals
- Non health professionals
- Implementers
- Policy makers

11. You as a health professional who is also in a priority group to be vaccinated, how did you feel after receiving the allocation/vaccines?
12. How do you think getting a COVID-19 vaccine might change some aspects in your life?.
    i. Probe: See family and friends, going out in public (if so, what made you difficult to go before getting vaccinated? Ask about worries/stigma?
13. As you have seen that the COVID-19 vaccine is recommended by health care professionals. How has the Family and friends responded? community leaders? Other professionals in your network?
14. How can you describe the uptake of vaccines by health professionals and other stakeholders in this region/district/health facility?
    i. How can you comment on the reaction of people after been vaccinated?
    ii. Any reported adverse events
    ii. Any reported admission?
15. How much do you trust the health care worker who will give you the vaccine?
    a. If vaccinated, what made it easy for you to do it? If not, then, what do you think will make it easy for you to get vaccinated?
16. What are the main factors promoting or hindering uptake of vaccines?
17. What do you think should be done to increase the uptake of vodid19 vaccine?
18. Do you have anything else regarding COVID-19 Vaccine that you would like to share with me?

THANK YOU FOR YOUR TIME TO PARTICIPANTE IN THIS INTERVIEW

Appendix 2

TOPIC GUIDE FOR FOCUS GROUP DISCUSSIONS AND GROUP DISCUSSION WITH HEALTH CARE WORKERS AT DISTRICT HOSPITALS AND HEALTH CENTERS

Introduction

Thank you for your willingness and voluntarily accepting to participate in this study. As you are already aware you will take part in the focus group discussion/group discussion to share your perspectives and experiences on COVID-19 and the respective responses in our setting. Before starting our discussion let us agreed on the logistics and ground rules:

- Focus group will last about one hour and one hour and a half (90 minutes)
- Feel free to move around to share your ideas as it is important for us to hear everyone's ideas and opinions
- Everyone should participate however only one person talks at a time
- There is no right or wrong answers to topics discussed so feel free to express your feelings about the issue being discussed
- The session will be audio recorded to accurately capture what we discuss
- Turn off or silence cell phones.
- No judgment or discussion of other's ideas
- Ideas (contributions) are anonymous, do not mention your names, rather use the numbers we gave you.
- Enjoy the discussions

COVID19 experiences

1. We are aware that similar to other parts around the world, our country experienced several waves of COVID-19 since early 2020. Can you share with us your experiences of the COVID-19?
   Probes
   - Stakeholder responses (government officials, professionals, community members)
2. As health care providers in this health facility, considering the different waves of covid 19, how risk do you see yourself to be infected with COVID-19?
   Probe
   - Any exposures with covid 19 in any of the waves?
   - How safe is your workplace to protect you from COVID-19?
3. What do you think about the risk that you could give COVID-19 to your patients? (Probe for experiences in all COVID-19)
4. What impacts have you noted in health facility functioning after having care providers who became infected and fall sick of COVID-19?
   Probes
   - Work motivation
   - Efficiency in performance
   - ? quality of care
5. Being health care workers, how are you usually treated by others in the community?
   Probe: Have you noticed anything different in how you're treated since the pandemic?

Introduction of COVID-19 vaccines

6. What have you heard about the COVID-19 vaccine?

Probe: encourage participants to express all that have heard about vaccines

7. Can you tell us on the sources of information about the COVID-19 vaccines?
   Probe: How credible were the sources? Which sources do you trust more? Why?
8. What can you say as positive sides about the vaccines that are currently available in the world?
   Probes :
   Which vaccines do you know?
   What do you think about the COVID-19 vaccines that are currently available in the country?
9. What are your thoughts about the safety of the vaccine? (Probe different types)
10. What are your thoughts about the effectiveness of COVID-19 vaccines (probe different types)? How much trust do you have on these vaccines? And why?

Availability and administration of COVID-19 vaccines in Tanzania

11. Health care workers are among the priority groups for COVID-19 vaccines in Tanzania, how do you feel about that and why?

Probes:

- Can you comment on health workers uptake of the COVID-19 vaccines?
- How available are vaccines in this facility/hospital?

11. As health care providers in this health facility, what are the general responses from peoples towards the vaccines currently administered?
    - Other health care workers
    - Non health professionals
    - People in the Community
    - Also probe: what do you think influences their responses
12. Generally, what are the misconception on vaccines by stakeholders at different levels
    - Community members
    - Health professionals
    - Non health professionals
    - Implementers
    - Policy makers
13. How can you describe the uptake of vaccines by health professionals and other stakeholders in health facility and the catchment population? How can you comment on the reaction of people after receiving vaccination?
    Any reported adverse events
    Any reported admission?
    Any worries and why
14. What are the factors promoting or hindering uptake of vaccines?
15. We know that the hesitance to vaccines lead to low acceptability hence low uptake of the COVID-19 vaccines.
16. What do you think should be done to clear the hesitance and ultimately increase the uptake of COVID-19 vaccine in the country?

THANK YOU FOR YOUR TIME TO TAKE PART IN THIS DISCUSSION
